# Molecular evolution of the polyamine oxidase gene family in Metazoa

**DOI:** 10.1186/1471-2148-12-90

**Published:** 2012-06-20

**Authors:** Fabio Polticelli, Daniele Salvi, Paolo Mariottini, Roberto Amendola, Manuela Cervelli

**Affiliations:** 1 Dipartimento di Biologia, Università “Roma Tre”, I-00146, Rome, Italy; 2 National Institute of Nuclear Physics, Roma Tre Section, I-00146, Rome, Italy; 3 CIBIO, Centro de Investigação em Biodiversidade e Recursos Genéticos, Campus Agrário de Vairão, 4485-661, Vairão, Portugal; 4 ENEA, CR Casaccia, BAS.BIOTEC MED, I-00123, Rome, Italy

## Abstract

**Background:**

Polyamine oxidase enzymes catalyze the oxidation of polyamines and acetylpolyamines. Since polyamines are basic regulators of cell growth and proliferation, their homeostasis is crucial for cell life. Members of the polyamine oxidase gene family have been identified in a wide variety of animals, including vertebrates, arthropodes, nematodes, placozoa, as well as in plants and fungi. Polyamine oxidases (PAOs) from yeast can oxidize spermine, N^1^-acetylspermine, and N^1^-acetylspermidine, however, in vertebrates two different enzymes, namely spermine oxidase (SMO) and acetylpolyamine oxidase (APAO), specifically catalyze the oxidation of spermine, and N^1^-acetylspermine/N^1^-acetylspermidine, respectively. Little is known about the molecular evolutionary history of these enzymes. However, since the yeast PAO is able to catalyze the oxidation of both acetylated and non acetylated polyamines, and in vertebrates these functions are addressed by two specialized polyamine oxidase subfamilies (APAO and SMO), it can be hypothesized an ancestral reference for the former enzyme from which the latter would have been derived.

**Results:**

We analysed 36 SMO, 26 APAO, and 14 PAO homologue protein sequences from 54 taxa including various vertebrates and invertebrates. The analysis of the full-length sequences and the principal domains of vertebrate and invertebrate PAOs yielded consensus primary protein sequences for vertebrate SMOs and APAOs, and invertebrate PAOs. This analysis, coupled to molecular modeling techniques, also unveiled sequence regions that confer specific structural and functional properties, including substrate specificity, by the different PAO subfamilies. Molecular phylogenetic trees revealed a basal position of all the invertebrates PAO enzymes relative to vertebrate SMOs and APAOs. PAOs from insects constitute a monophyletic clade. Two PAO variants sampled in the amphioxus are basal to the dichotomy between two well supported monophyletic clades including, respectively, all the SMOs and APAOs from vertebrates. The two vertebrate monophyletic clades clustered strictly mirroring the organismal phylogeny of fishes, amphibians, reptiles, birds, and mammals. Evidences from comparative genomic analysis, structural evolution and functional divergence in a phylogenetic framework across Metazoa suggested an evolutionary scenario where the ancestor PAO coding sequence, present in invertebrates as an orthologous gene, has been duplicated in the vertebrate branch to originate the paralogous *SMO* and *APAO* genes. A further genome evolution event concerns the *SMO* gene of placental, but not marsupial and monotremate, mammals which increased its functional variation following an alternative splicing (AS) mechanism.

**Conclusions:**

In this study the explicit integration in a phylogenomic framework of phylogenetic tree construction, structure prediction, and biochemical function data/prediction, allowed inferring the molecular evolutionary history of the *PAO* gene family and to disambiguate paralogous genes related by duplication event *(SMO* and *APAO*) and orthologous genes related by speciation events (*PAO*s, *SMO*s/*APAO*s). Further, while in vertebrates experimental data corroborate SMO and APAO molecular function predictions, in invertebrates the finding of a supported phylogenetic clusters of insect PAOs and the co-occurrence of two PAO variants in the amphioxus urgently claim the need for future structure-function studies.

## Background

**Figure 1:**
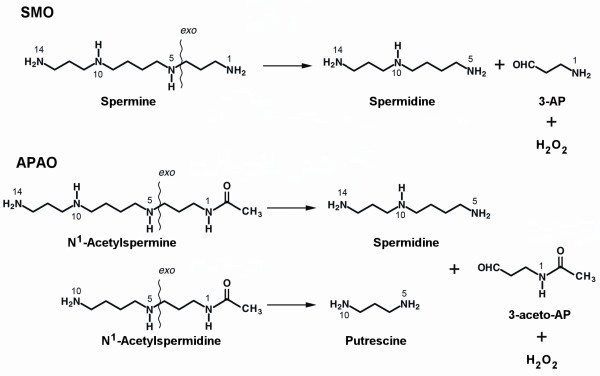
**Enzymatic reaction catalyzed by SMO and APAO proteins. **SMO oxidises the carbon on the *exo*-side of the N^5^-nitrogen of Spm, producing Spd, 3-aminopropanal (3-AP) and hydrogen peroxide (H_2_O_2_). APAO oxidises the carbon on the *exo*-side of the N^5^-nitrogen of N^1^Ac-Spm and N^1^Ac-Spd producing Spd and Put respectively, in addition to 3-aceto-aminopropanal (3-aceto-AP) and H_2_O_2_.

Polyamines (PA), such as spermine (Spm), spermidine (Spd) and putrescine (Put), are polybasic molecules ubiquitous in living organisms, with many important biological functions. These molecules directly affect cell growth, differentiation, and apoptosis by reversibly interacting with nucleic acids, regulating chromatin status and gene expression, and modulating ion-channels’ function and stability [1,2]. Polyamine oxidases (PAO) are flavin adenine dinucleotide- (FAD-) containing enzymes that catalyze the oxidation of polyamines. The substrate specificity and the nature of the oxidation products depend on the source of the enzymes. Generally, PAOs oxidizes Spm, N^1^-acetylspermine (N^1^-acetylSpm) and N^1^-acetylspermidine (N^1^-acetylSpd), but not Spd. On the other hand, in vertebrates Spm is directly oxidized by the cytosolic enzyme spermine oxidase (SMO, EC number 1.5.3.16), a flavoprotein characterized in the past as a human polyamine oxidase (PAO-h1) [3] and then subsequently named SMO [4,5]. Spm oxidation leads to the production of Spd, 3-aminopropanal and hydrogen peroxide (H_2_O_2_). While N^1^-acetylSpm and N^1^-acetylSpd are oxidized by the peroxisomal FAD-dependent enzyme N^1^-acetylpolyamine oxidase (APAO, EC number 1.5.3.11) to produce respectively Spd and Put, 3-aceto-aminopropanal and H_2_O_2_. Substrate oxidation modes of SMO and APAO are summarized in Figure 1.

In the last decades, these PA catabolic enzymes have been extensively characterized and it is well documented that both enzymes play an essential role in maintaining vertebrate PA homeostasis, which is mandatory for cellular life [2,6-9]. Unfortunately, repeated attempts from independent labs to obtain SMO and APAO crystals suitable for X-ray diffraction studies failed [A. Mattevi, personal communication; A. Fiorillo and A. Ilari, personal communication]. The only structural data available for these enzymes are those derived from molecular modeling studies of mouse SMO [5,10,11] and mouse APAO [12] which indicated a very similar active site environment for the two proteins, making it difficult to rationalize their different substrate specificity and sensitivity to small molecule inhibitors [13]. On the contrary, the crystal structure of the *Saccharomyces cerevisiae* PAO (FMS1) has been obtained and its biochemical characterization proved that it is able to oxidize Spm, N^1^-acetylSpm and N^1^-acetylSpd [14,15]. Since the yeast PAO is capable to catalyse the oxidation of both acetylated and non-acetylated polyamines, and in vertebrates these functions are addressed by two specialized polyamine oxidase subfamilies (APAO and SMO), it can be hypothesized an ancestral reference for PAO enzymes and a paralogous relationships between APAOs and SMOs. Yet, we still have a limited knowledge on the structural and functional diversity of metazoans polyamine oxidases and their evolutionary history has never been studied.

In this study we developed a phylogenomic framework [16] to investigate the phylogenetic relationships, the structural evolution and the functional divergence among polyamine oxidases proteins in animals. We identified, through an exhaustive BLASTP search strategy all the available proteins homologous to SMO and APAO enzymes and we assembled a comprehensive multiple amino acid sequences alignment including 76 polyamine oxidases from all the vertebrate classes and main invertebrate phyla. Phylogenetic analysis allowed inferring an evolutionary scenario where the ancestor PAO coding sequence, present in invertebrates as an orthologous gene, has been duplicated in the vertebrate branch to originate the paralogue SMO and APAO genes. Overlaying the tree topology with data from molecular structure modelling and biochemical function data/prediction, we traced along the evolutionary tree the processes behind the origin of the functional and structural diversity found in polyamine oxidase proteins. Finally, the presence of the alternative SMO protein isoform [10,17,18] was confirmed in all the placental mammals analysed, suggesting that in this group a mechanism of alternative splicing (AS) allowed a further increase of the structural and functional variation of the SMO proteins.

## Results and discussion

### Clustering SMO, APAO and PAO homologous proteins

An exhaustive species-specific BLASTP search of PAO homologs was carried out in the available databases using the following threshold values: E-value = 1×10^-10^, > 30% sequence identity over at least 50% of the ORF length (see section Methods for details). These searches enabled us to retrieve 76 homologous PAO proteins from the baker’s yeast (*Saccharomyces cerevisiae*) and 52 animal taxa (Table 1), including nematode (*Caenorhabditis elegans*), placozoa (*Trichoplax adhaerens*), echinoderms (*Strongylocentrotus purpuratus* and *Nematostella vectensis*), arthropods (*Pediculus humanus corporis*, *Tribolium castaneum*, *Drosophila melanogaster*, *Anopheles gambia*, *Nasonia vitripennis*, and *Apis mellifera*), and chordates such as urochordates (*Ciona intestinalis*), cephalochordates (*Branchiostoma floridae*), and vertebrates: fishes (*Tetraodon nigroviridis*, *Takifugu rubripes*, *Oryzias latipes*, *Danio rerio*, and *Gasterosteus aculeatus*), amphibians (*Xenopus laevis* and *X. tropicalis*), reptile (*Anolis carolinensis*), birds (*Gallus gallus*, *Taeniopygia guttata*, and *Meleagris gallonavo*), monotremate mammal (*Ornithorhyncus anatinus*), marsupial mammals (*Monodelphis domestica*, and *Macropus eugenii*), and placental mammals (*Procavia capensis*, (*Loxodonta africana*, *Microcebus murinus*, *Rattus norvegicus*, *Mus musculus*, *Cavia porcellus*, *Dipodomys ordii*, *Tarsius syrinchtae*, *Callithrix jacchus*, *Macaca fascicularis*, *Nomascus leucogenys*, *Gorilla gorilla*, *Pan troglodytes*, *Pongo pygmaeus*, *Pongo abelii*, *Homo sapiens*, *Equus caballus*, *Pteropus vampyrus*, *Myosotis lucifugus*, *Felis catus*, *Canis familiaris*, *Ailuropoda melanoleuca*, *Sus scrofa*, *Tursiops truncatus*, *Bos taurus,* and *Oryctogalus cuniculus*).

**Table 1:** Polyamine oxidase proteins sequences used in this study

**Organism and acronym [Enzyme]**	**Evidence**	**Accession number**
**[Spermine oxidase (SMO, EC number 1.5.3.16)]**		
*Ailuropoda melanoleuca* giant panda (Am)	predicted protein	[GenBank:EFB25976]
*Anolis carolinensis* green anole (An)	predicted protein	[ID:ENSACAP00000000096]
*Bos taurus* cattle (Bt)	protein	[Swiss-Prot:Q865R1]
*Callithrix jacchus* pygmy marmoset (Cj)	predicted protein	[ID:ENSCJAP00000039556]
*Canis familiaris* dog (Cf)	predicted protein	[GenBank:XP_860548]
*Cavia porcellus* guinea pig (Cp)	predicted protein	[ID:ENSCPOP00000015071]
*Danio rerio* zebrafish (Dr)	protein	[Swiss-Prot:Q6NYY8]
*Dipodomys ordii* kangaroo rat (Do)	predicted protein	[ID:ENSDORP00000002003]
*Equus caballus* horse (Eq)	predicted protein	[GenBank: XP_001495419]
*Felis catus* cat (Fc)	predicted protein	[ID:ENSFCAP00000002991]
*Gallus gallus* chicken (Gg)	predicted protein	[GenBank:XP_420872]
*Gasterosteus aculeatus* three-spined stickleback (Ga)	predicted protein	[ID:ENSGACP00000023283]
*Gorilla gorilla* gorilla (Go)	predicted protein	[ID:ENSGGOP00000018695]
*Homo sapiens* human (Hs)	protein	[Swiss-Prot:Q99K82]
*Loxodonta africana* elephant (La)	predicted protein	[ID:ENSLAFP00000010450]
*Macaca fascicularis* macaque monkey (Mf)	predicted protein	[GeneBank:BAE88223]
*Macropus eugenii* wallaby (Me)	predicted protein	[ID:ENSMEUP00000000797]
*Microcebus murinus* lemur mouse (Mu)	predicted protein	[ID:ENSMICP00000006250]
*Monodelphis domestica* grey short-tailed opossum (Md)	predicted protein	[GenBank:XP_001380279]
*Mus musculus* house mouse (Mm)	protein	[Swiss-Prot:NP_663508]
*Myosotis lucifugus* microbat (Ml)	predicted protein	[ID ENSMLUP00000014140]
*Nomascus leucogenys* gibbon (Nl)	predicted protein	[ID:ENSNLEG00000007689]
*Ornithorhyncus anatinus* platypus (Oa)	predicted protein	[GenBank:XP_001516006]
*Oryctogalus cuniculus* rabbit (Oc)	predicted protein	[ID:ENSOCUP00000006931]
*Oryzias latipes* medaka (Ol)	predicted protein	[ID:ENSORLP00000007986]
*Pan troglodytes* chimpanzee (Pt)	protein	[Swiss-Prot:XP_514493]
*Pongo abelii* sumatra orangutan (Pa)	predicted protein	[GenBank:XP_002830111]
*Rattus norvegicus* rat (Rn)	protein	[Swiss-Prot:XP_001079707]
*Sus scrofa* pig (Ss)	predicted protein	[GenBank:XR_04566]
*Taeniopygia guttata* zebra finch (Tg)	predicted protein	[GenBank:XP_002189301]
*Takifugu rubripes* japanese pufferfish (Tr)	predicted protein	[ID:ENSTRUP00000003466]
*Tarsius syrinchtae* tarsier (Ts)	predicted protein	[ID:ENSTSYP00000002870]
*Tetraodon nigroviridis* green spotted pufferfish (Tn)	predicted protein	[ID:ENSTNIP00000001941]
*Tursiops truncatus* dolphin (Tt)	predicted protein	[ID:ENSTTRP00000009415]
*Xenopus laevis* african clawed frog (Xl)	protein	[Swiss-Prot:Q6INQ4]
*Xenopus tropicalis* western clawed frog (Xt)	protein	[Swiss-Prot:Q28C17]
**[Acetylpolyamine oxidase (APAO, EC number 1.5.3.11)]**		
*Anolis carolinensis* green anole (An)	predicted protein	[XP_003225445]
*Bos taurus* cattle (Bt)	protein	[Swiss-Prot:Q865R1]
*Callithrix jacchus* pygmy marmoset (Cj)	predicted protein	[ID:ENSCJAP00000009627]
*Cavia porcellus* guinea pig (Cp)	predicted protein	[ID:ENSCPOP00000010900]
*Danio rerio* zebrafish (Dr)	predicted protein	[GenBank**:**XP_690593]
*Equus caballus* horse (Eq)	predicted protein	[ID:ENSECAP00000000093]
*Gallus gallus* chicken (Gg)	predicted protein	[ID ENSGALP00000005619]
*Gasterosteus aculeatus* three-spined stickleback (Ga)	predicted protein	[GenBank: BT027282]
*Gorilla gorilla* gorilla (Go)	predicted protein	[ID:ENSGGOP00000004628]
*Homo sapiens* human (Hs)	protein	[Swiss-Prot:Q6QHF9-1**]**
*Loxodonta africana* elephant (La)	predicted protein	[ID:ENSLAFP00000007186]
*Macaca mulatta* macaque monkey (Ml)	predicted protein	[ID:ENSMMUP00000008331]
*Macropus eugenii* wallaby (Me)	predicted protein	[ID:ENSMEUP00000004459]
*Monodelphis domestica* short-tailed opossum(Md)	predicted protein	[ID:ENSMODP00000013113**]**
*Mus musculus* house mouse (Mm)	protein	[Swiss-Prot:Q4GX45]
*Oryzias latipes* medaka (Ol)	predicted protein	[ID:ENSORLP00000011447]
*Pongo pygmaeus* orangutan (Pp)	predicted protein	[ID:ENSPPYP00000003262]
*Procavia capensis* hyrax (Pc)	predicted protein	[ID:ENSPCAP00000005028]
*Pteropus vampyrus* megabat (Pv)	predicted protein	[ID:ENSPVAG00000002682]
*Rattus norvegicus* rat (Rn)	protein	[Swiss-Prot:Q7TPJ4]
*Taeniopygia guttata* zebra finch (Tg)	predicted protein	[GenBank:XP_002186801]
*Takifugu rubripes* japanese pufferfish (Tr)	predicted protein	[ID:ENSTRUP00000035024]
*Tetraodon nigroviridis* green spotted pufferfish 1 (Tn1)	predicted protein	[ID:ENSTNIP00000019636]
*Tetraodon nigroviridis* green spotted pufferfish 2 (Tn2)	predicted protein	[ID:ENSTNIP00000002385]
*Tursiops truncatus* dolphin (Tt)	predicted protein	[ID:ENSTTRP00000014996]
*Xenopus laevis* african clawed frog (Xl)	protein	[Swiss-Prot: Q5U4L6]
**[Polyamine oxidases PAOs]**		
*Anopheles gambia* malaria mosquito (Ag)	predicted protein	[XP_312316.3]
*Apis mellifera* honey bee (Am)	predicted protein	[GenBank:XP_001122522]
*Branchiostoma floridae* amphioxus florida lancelet 1 (Bf1)	predicted protein	[GenBank:XP_002225568]
*Branchiostoma floridae* amphioxus florida lancelet 2 (Bf2)	predicted protein	[GenBank:XP_002606976]
*Caenorhabditis elegans* nematode roundworm (Ce)	predicted protein	[GenBank:NP_001023872]
*Ciona intestinalis* sea squirt (Ci)	predicted protein	[XP_002132119]
*Drosophila melanogaster* fruit fly (Dm)	protein	[Swiss-Prot:Q9VHN8]
*Nasonia vitripennis* jewel wasp (Nv)	predicted protein	[XP_001599761]
*Nematostella vectensis* starlet sea anemone (Ne)	predicted protein	[GenBank:XP_001626025]
*Pediculus humanus corporis* human body louse (Ph)	predicted protein	[GenBank:EEB13427]
*Saccharomyces cerevisiae* baker’s yeast (Sc)	protein	[GenBank:YDL174C]
*Strongylocentrotus purpuratus* purple urchin (Sp)	predicted protein	[GenBank:XP_001195328]
*Tribolium castaneum* red flour beetle (Tc)	predicted protein	[GenBank:XP_971067]
*Trichoplax adhaerens* tablet animal-Placozoa (Ta)	predicted protein	[GenBank:XP_002107802]

List of sequences included in the phylogenetic, structural and functional analyses and their corresponding accession numbers.

Among the 75 homologous PAO sequences found in Metazoa, 13 sequences are annotated as invertebrate PAO proteins, 36 as vertebrate SMO proteins, and 26 as vertebrate APAO proteins. In few cases among arthropods we found additional sequences which showed some similarity with PAOs, but their overall identity with PAOs was lower than 30% and it was not possible to obtain a reliable alignment along the entire length. Thus, we consider these sequences as non-homologs to PAOs. The lower number of APAO sequences retrieved compared with SMO sequences is due to the availability of sequences in Genbank rather than to the absence in certain vertebrate taxa of APAO proteins.

### Phylogeny and evolution of the polyamine oxidase family

**Figure 2:**
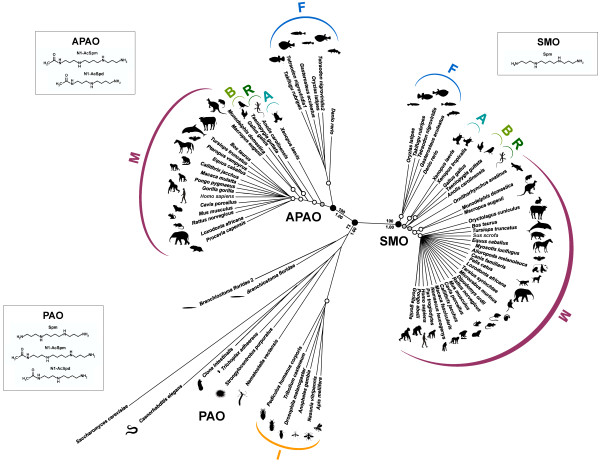
**The evolutionaty tree of the the polyamine oxidase proteins. **The evolutionary history tree of the SMO, PAO, and APAO polyamine oxidase proteins in Metazoa as inferred by using the Maximum Likelihood method based on the JTT model with a proportion of invariable sites (I) and gamma-distributed rates across sites (G) (Bayesian tree showed identical topology at the main nodes; data not shown). The analysis involved 76 amino acidic sequences with the yeast sequence used as outgroup. In correspondence of the main nodes (black circles) the bootstrap support (BS) and Bayesian posterior probabilities (BPP) values are reported (above: BS; below: BPP). The support for the secondary nodes is reported as white circles (BS = 100; BPP = 1.00) and grey circles (BS ranging from 90 to 99; BPP = 1.00). The tree is drawn to scale, with branch lengths measured in the number of substitutions per site. Main monophyletic groups are indicated as follow: M = mammals; B = birds; R = reptiles; A = amphibians; F = fishes; I = insects.

The phylogenetic relationship among the polyamine oxidase proteins as inferred by the Maximum Likelihood and Bayesian trees were consistent at the main nodes and revealed a basal position of all the PAO enzymes present in invertebrates relative to vertebrate SMOs and APAOs (Figure 2). Polyamine oxidases from invertebrates such as placozoa, nematodes, echinoderms, and urochordates do not constitute a monophyletic assemblage, while PAOs from insects, although fairly differentiated from each other, all shared a common ancestor and constitute a derived and supported clade within the PAO group. However, the lack of support for other PAO groups can be due to a sparse taxon sampling [19]. The two PAO variants sampled in the cephalochordate amphioxus are basal to the dichotomy between two well supported monophyletic clades including, respectively, all the SMOs and APAOs from vertebrates.

These results provide evidence that SMO and APAO subfamilies originate from a duplication event from a PAO-like gene ancestor, while gene speciation accounts for their ubiquitous occurrence in vertebrates. Moreover, the obvious difference in substrate specificity between SMO and APAO enzymes ([3,20] and results from this study) indicates that after the duplication these gene subfamilies underwent divergent evolution and functional specialization.

Within both SMO and APAO subfamilies, ortholog sequences show substantial diversity and divergence among and within vertebrate classes. Fish polyamine oxidases constitute a distinct clade from that of tetrapods in which polyamine oxidases from amphibians, reptiles, birds and mammals cluster in different protein lineages, although phylogenetic relationships between these protein lineages are largely unresolved both in the SMO and APAO branches (Figure 2). Within the mammals a further evolution of both SMO and APAO polyamine oxidase proteins can be traced along the evolution of Monotremata, Marsupialia, and between major groups of Placentalia. Indeed, the APAOs of Afrotheria (Proboscidea and Hyracoidea), Cetartiodactyla (Artiodactyla and Cetacea), Rodentia and Primates, as well as SMOs of Carnivora, Cetartiodactyla (Artiodactyla and Cetacea), and Primates, constituted monophyletic supported clades ([21,22] for taxonomic reference). Thus, the phylogenetic relationships among both SMO and APAO orthologs strictly mirror the phylogenetic relationships within vertebrates, suggesting that these SMO and APAO proteins evolved throughout the speciation events in vertebrates.

The co-occurrence of SMO and APAO enzymes in all the vertebrates suggests that their specific functions evolved earlier than vertebrates. Besides, the presence of two related PAOs in the cephalochordate amphioxus suggests that the duplication event from which they originated could have even pre-dated the Chordate radiation. According to this hypothesis, we would have expected two PAO gene copies also in the urochordate taxon here analysed, *Ciona intestinalis*, as recent molecular phylogenetic studies place cephalochordates as the basal group within the phylum Chordata, with vertebrates and urochordates diverging later [23,24]. However, several studies demonstrated that the urochordate genomes have lost many genes respect to cephalochordate and vertebrate genomes [25-27], thus the presence of a single PAO gene copy in *Ciona intestinalis* could be due to a secondary gene loss. An alternative and equally parsimonious hypothesis could be that a lineage-specific duplication of the *PAO* gene occurred in the amphioxus and an independent duplication followed by functional divergence arose in the ancestor of the vertebrates. In amphioxus several genes have gone through lineage-specific duplications relative to the chordate ancestor, for example some genes belonging to the opsin and to the innate immunity receptor groups are organized in gene families that have dramatically expanded copy number of homologs (reviewed in [28]; see also [27,29,30]). These findings are not surprising given that amphioxus has been evolving from the common ancestor of cephalochordates and vertebrates for over 550 Myr. In the case of the two *PAO* gene copies of amphioxus, due to their low amino acid sequence similarity (37%), these genes likely arose by an ancient duplication event. However, a broad comparative genome analysis and structure-function studies on chordates PAOs are required before it will be possible to understand whether this duplication pre-dated the Chordate radiation or it is specific to the amphioxus lineage and how the functions of the two *PAO* genes found in such chordates were partitioned during evolution.

### Structural and functional properties of invertebrate PAOs

Polyamine oxidases from invertebrates do not constitute a monophyletic assemblage in our phylogenetic reconstruction and no clear pattern of residues conservation can be evidenced in the active site region (Additional file 1: Figure S1). This finding is consistent with the hypothesis that invertebrate PAOs have broad substrate specificity as indeed it has been demonstrated for the best characterized member of this proteins group, the *Saccharomyces cerevisiae* PAO (FMS1). In fact, FMS1 is capable of oxidizing Spm, N^1^-acetylSpm, N^1^-acetylSpd, and N^8^-acetylSpd, but not Spd [15].

**Figure 3:**
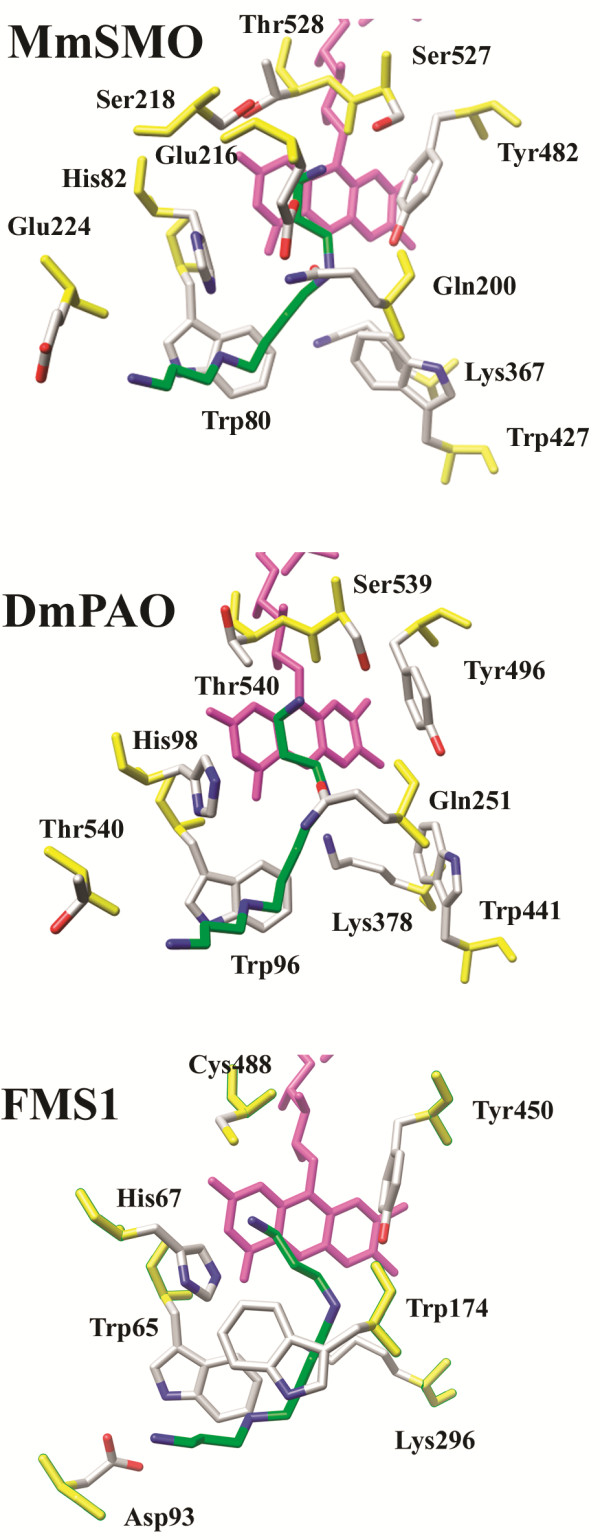
**Molecular models of the active site region of SMO and PAO. **Schematic representation of the active site region of mouse SMO (MmSMO), *Drosophila melanogaster *PAO (DmPAO) and *Saccharomyces cerevisiae *PAO (FMS1) in complex with the substrate Spm. MmSMO and DmPAO complexes with Spm have been obtained by molecular modelling ([11] and this work, respectively) while the structure of the FMS1-Spm has been determined experimentally [14]. For the sake of clarity, the FAD cofactor is coloured in purple, backbone atoms in orange and Spm carbon atoms in green.

Interestingly, according to the phylogenetic analysis (Figure 2), PAOs from insects are all derived from a common ancestor and constitute a well-defined and supported clade within the PAOs’ branch. To investigate the structural and functional properties of insect PAOs, a molecular model of *Drosophila melanogaster* PAO (DmPAO) has been built by homology using the three-dimensional structure of maize PAO, the closest homolog found in the Protein Data Bank [31], as a template. Unexpectedly, inspection of the active site region of DmPAO evidenced a stronger structural similarity with the SMOs active site than with that of the invertebrate PAO and of FMS1 (Figure 3). In particular, all the residues involved in substrate binding in SMOs are conserved in DmPAO with the exception of Glu224 substituted with a Thr residue which, nonetheless, can potentially interact with the substrate primary amino group (Figure 3). In addition, comparative analysis of the amino acid sequences of insects PAOs and mammalian SMOs and APAOs indicates that the polar active site pocket hypothesized to be responsible for the substrate specificity of SMOs (see the next section) has very similar properties in insect PAOs. This observation suggests that substrate specificity of insect PAOs may not be as broad as that of FMS1. However, our *in silico* analysis of DmPAO does not allow to draw a reliable conclusion on this respect and for a deep understanding of the functional properties of insects PAOs a structural and biochemical characterization of these enzymes is required.

In contrast with other invertebrates, the genome of the amphioxus encodes two PAO-isologs. As previously discussed, two PAO copies are only found in vertebrates and in the amphioxus suggesting that the duplication of the ancestral PAO gene predated the Chordate radiation. Thus, the knowledge of the structural and biochemical properties of the amphioxus’ PAO variants is crucial for understanding whether the duplication of the ancestral PAO gene and the functional divergence between the resulting paralogs were strictly coupled or not during the evolution of the polyamine oxidase subfamilies. Although the biochemical function of the two amphioxus’ PAOs is completely unknown, as they show a substantial sequence divergence (see Additional file 1: Figure S1) it is plausible the hypothesis of an early functional divergence between them from an ancestral PAO-like function.

### Structural and functional properties of SMOs and APAOs

**Figure 4:**
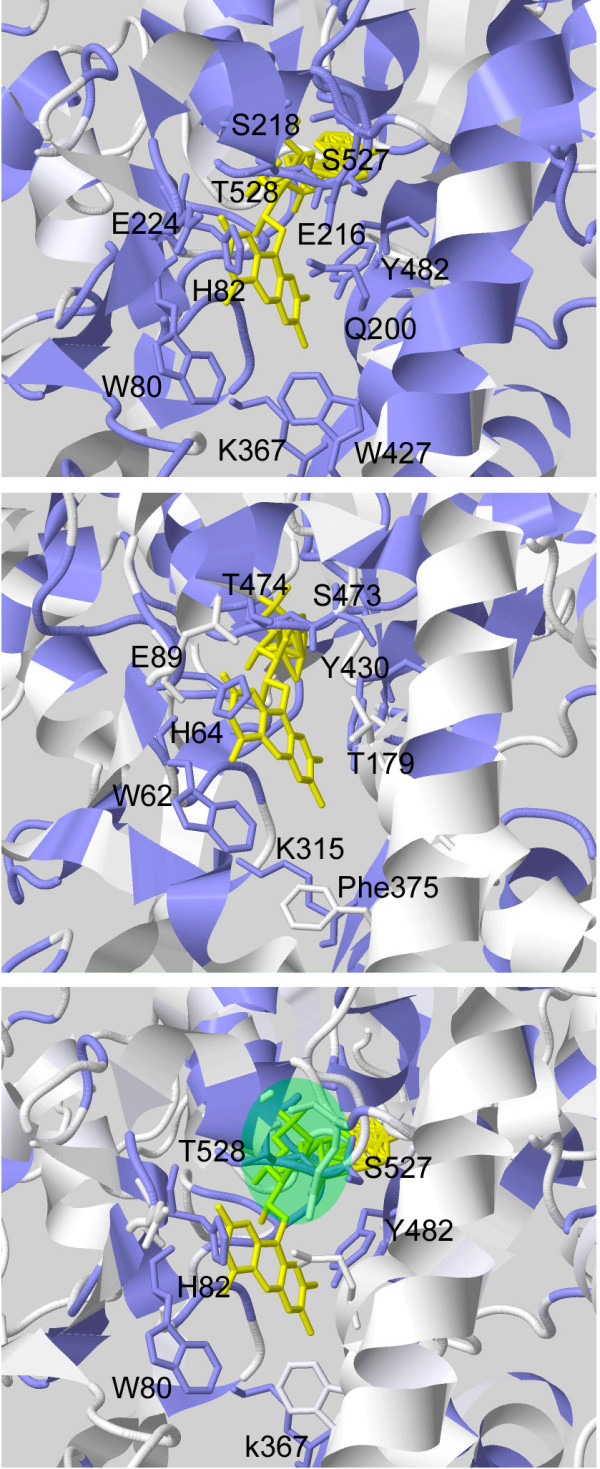
**Three-dimensional view of SMOs and APAOs sequence conservation. **Structure-based views of the amino acid sequence conservation in the active site regions of vertebrate SMOs and APAOs. Protein regions coloured in blue correspond to residues conserved in at least 90% of the amino acid sequences analysed. Top panel: Amino acid sequence conservation in the SMO family mapped onto the structural model of mouse SMO [11]. Middle panel: Amino acid sequence conservation in the APAO family mapped onto the structural model of mouse APAO [12]. Bottom panel: Amino acid sequence conservation in the vertebrate PAO family (SMO and APAO sequences combined) mapped onto the structural model of mouse SMO. The green ellipse indicates the location of the polar pocket made up SMOs by residues Glu216 and Ser218 (numbering of the mouse enzyme), which are substituted by aliphatic amino acids in APAOs. The figure was generated using Jalview [32].

Molecular modeling allowed mapping the sequence conservation of SMOs and APAOs onto the three-dimensional structure of a previously published molecular model of these enzymes [11,12]; see Methods section and to identify critical amino acids of the active site regions. The three-dimensional models displayed in Figure 4, shows that the specificity of the SMO enzymes results in a very high degree of conservation of all the residues building up the active site cavity. In detail, polar residues (His82, Gln200, Glu224, Tyr482, Ser527, Thr528) and hydrophobic residues (Trp80 and Trp427) thought to interact with Spm, as well as the catalytically crucial Lys367 [11], are all conserved in more than 90% of the sequences analysed. Interestingly, also residues Glu216 and Ser218, which form a pocket on one side of the active site, are almost strictly conserved in all SMO sequences analysed. In the case of APAO, instead, there seems to be a lower evolutionary pressure towards strict conservation of the active site residues, the only residues conserved in more than 90% of the sequences analysed being Trp62, His64, Tyr430, Ser473 and Thr474 (ortholog to SMO residues Trp80, His82, Tyr482, Ser527 and Thr528), besides the catalytically important Lys315 (Figure 4). It is worthwhile to note that all the residues conserved in APAOs are shared with SMOs and thus the different specificity of the two enzymes appears difficult to rationalize. From this viewpoint, an interesting observation is that the SMOs strictly conserved residues Glu216 and Ser218 are substituted by hydrophobic residues in APAOs (typically with a Leu and an Ala residue, respectively) making the corresponding active site pocket of APAO fit to host a hydrophobic group rather than a charged one. Indeed, docking of N^1^-acetylSpm within the mouse SMO active site indicates that the methyl group of this molecule would be placed in the polar pocket made up by Glu216 and Ser218, making energetically unfavourable the binding of N^1^-acetylSpm within the SMO active site [11]. On the contrary, binding of Spm to APAO would lead to positioning of one of the charged terminal amino groups of this molecule within the Leu-Ala hydrophobic pocket, making energetically unfavourable the binding of Spm within the APAO active site [11].

### Mammalian SMO “long” isoform

SMO genes are able to increase the structural and functional variation of the corresponding proteins following an alternative splicing (AS) mechanism. Indeed, alternative splicing isoforms of SMO have been described in human and mouse, defined as “major” (i.e., SMO1 and SMOα for human and mouse proteins, respectively) and “long” isoforms (i.e., SMO5 and SMOμ for human and mouse proteins, respectively) [17,18]. The long isoforms possess an additional exon (exon VIa in mouse SMO) which is responsible for their nuclear localization and thus defined as Nuclear Domain B (NDB) [10]. The nuclear localization of the long isoforms requires also a second domain termed Nuclear Domain A (NDA), which is an internal amino acid stretch of the common exon V also present in the major isoforms [10].

**Figure 5:**
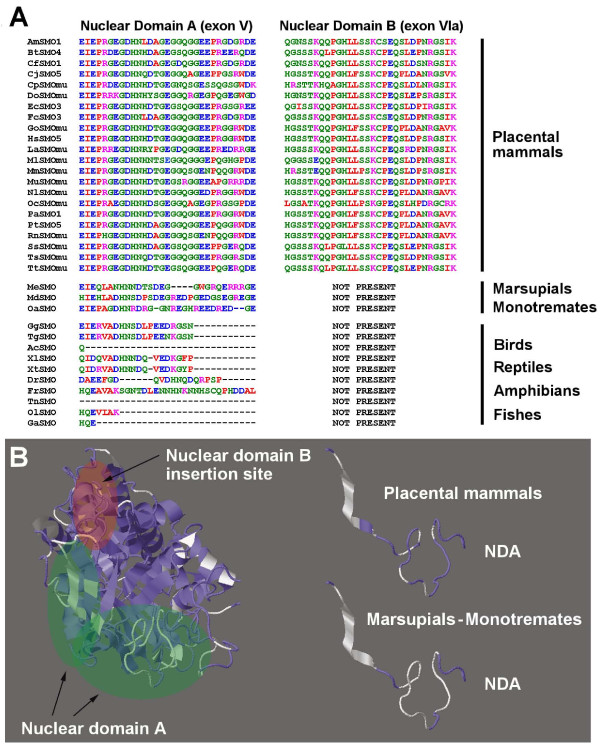
**Amino acid sequence alignment and structure-based view of SMO isoforms. A**) Amino acid sequence alignment of the regions corresponding to Nuclear Domain A (NDA) and B (NDB) of SMO long isoforms. For acronyms and isoform numbering see Table 2. **B**) Structure-based view of the amino acid sequence conservation in SMOs (left) and in the NDA of placental mammals as opposed to marsupials and monotremates (right). Protein regions coloured in blue correspond to residues conserved in at least 90% of the amino acid sequences analysed.

To investigate the presence in mammalian genomes of these alternative splicing SMO isoforms and to analyse their sequence conservation, we performed a search in the public databases based on exon VIa sequence annotation that enabled us to retrieve 22 sequences corresponding to the long SMO isoform Table 2. All these sequences were identified in genomes from placental mammals. Surprisingly, no corresponding alternative splicing variant was found in marsupials (wallaby and gray short-tailed opossum) and monotremates (platypus) *SMO* genes (Figure 5) which also showed a lack of sequence conservation in the NDA regions. The absence of sequence conservation of the NDA and the lacking of the NDB was also confirmed in the *SMO* genes of birds, reptiles, amphibians and fishes (Figure 5). On the contrary, both the NDA and the NDB displayed a high degree of sequence conservation across all the placental mammals analysed, suggesting common structural and functional properties. Given the ubiquitous occurrence of these SMO isoforms in placental mammals, their high degree of sequence conservation, and their absence in marsupials and monotremates, a role of SMO isoforms in placenta development can be postulated.

**Table 2:** SMO sequences used in the analysis of the additional exon VIa

**Organism and acronym**	**Accession number**	**Isoform number**
*Ailuropoda melanoleuca* (Am)	[GL195116]	isoform 1
*Bos taurus* (Bt)	[XM_864722, XP_869815]	isoform 4
*Canis familiaris* (Cf)	[XP_542910]	isoform 1
*Callithrix jachus* (Cj)	[ENSCJAP00000039547]	isoform mu
*Cavia porcellus* (Cp)	[ENSCPOG00000024093]	isoform mu
*Dipodomys ordii* (Do)	[ENSDOR00000002138]	isoform mu
*Equus caballus* (Ec)	[XP_001495489]	isoform 3
*Felis catus* (Fc)	[ENSFCAP00000002991]	isoform mu
*Gorilla gorilla* (Go)	[ENSGGOP00000010403]	isoform mu
*Homo sapiens* (Hs)	[ABM01872]	isoform 5
*Loxodonta africana* (La)	[ENSLAF00000022779]	isoform mu
*Macropus eugenii* (Me)	[ENSMEUG00000000861]	no isoform
*Microcebus murinus* (Mu)	[ENSMICG00000006861]	isoform mu
*Monodelphis domestica* (Md)	[XM_001380242]	no isoform
*Mus musculus* (Mm)	[AJ567473]	isoform mu
*Myosotis lucifugus* (Ml)	[ENSMLUG00000015515]	isoform mu
*Nomascus leucogenys* (Nl)	[ID:ENSNLEG00000007689]	isoform mu
*Ornithorhyncus anatinus* (Oa)	[XP_001516006,XM_001515956]	no isoform
*Oryctogalus cuniculus* (Oc)	[ENSOCUG00000008024]	isoform mu
*Pan troglodytes* (Pt)	[XP_001163910]	isoform 5
*Pongo abelii* (Pa)	[XP_002830110]	isoform 1
*Rattus norvegicus* (Rn)	[XM_218704]	isoform mu
*Sus scrofa* (Ss)	[AK236942]	isoform mu
*Tarsius syrinchtae* (Ts)	[ENSTSYG00000003146]	isoform mu
*Tursiops truncatus* dolphin (Tt)	[GeneScaffold_412:9956:27593:1]	isoform mu

List of mammalian sequences utilized in the SMO long isoform analysis and their corresponding accession numbers.

These results suggested that SMO isoforms can be considered as a relatively recent evolutionary acquisition of (placental) mammals, where *SMO* gene increased its functional variation following an AS mechanism. Therefore, in PAOs, besides gene duplication, the AS mechanism is a further evolutionary mechanism which efficiently amplifies the gene variation and relative functional differentiation by producing different transcripts through a splicing machinery, even though the gene remains a single copy [33,34].

## Conclusions

In the present study the explicit integration of phylogenetic reconstruction, homology-based structure prediction, and biochemical function data/prediction allowed understanding the molecular evolution of the polyamine oxidase gene family in Metazoa and provided a deeper insight into the structure(s)-function(s) of individual members. Gene duplication and speciation were identified as the evolutionary processes by which the functional and structural diversity observed in this protein family originated. Through gene duplication from an ancestral PAO-like gene coding for a polyamine oxidase with broad substrate specificity, the vertebrate SMO and APAO subfamilies evolved related but distinct functions. On the other hand, gene speciation (and also alternative splicing in the case of SMO of placental mammals) accounts for the protein diversity observed within each one of these two subfamilies.

The phylogenomic approach here employed allowed to trace along the evolutionary tree the acquisition of specific biochemical functions by the SMO and APAO subfamilies. However, while for vertebrates a representative sampling of SMO and APAO protein sequences is available and experimental data corroborate their predicted molecular function, for invertebrates PAOs few protein sequences and no structural and biochemical data are available, thus preventing a conclusive inference of their function and relationships. In this respect, the finding of the monophyletic clade of insect PAOs, and the occurrence of two PAO variants in the cephalochordate amphioxus, provide a guide for future structure-function studies aimed at clarifying the biochemical function of PAOs in invertebrates and the timing of the duplication event which originated the vertebrate SMO and APAO subfamilies.

## Methods

### Protein and gene sequence homology search and retrieval

Annotated protein databases such as UniProt (http://www.ebi.ac.uk/uniprot/) and PFAM (http://www.sanger.ac.uk/Sofware/Pfam/) and major sequence repositories such as the National Center for Biotechnology Information (http://www.ncbi.nlm.nih.gov/) provide direct access to many known, full-length SMO, APAO and PAO protein sequences. Amino acid sequences from animal taxa and from the yeast were obtained using a combination of queries based on key terms and BLASTP [35] searches. Whenever possible, sequences whose enzymatic activities had been previously verified have been used as query sequences in all blast searches. Additional SMO, APAO and PAO predicted protein sequences were retrieved from currently sequenced and unfinished genomes at the Ensembl (http://www.ensembl.org/index.html) database. A BLAST search was conducted (search with the same dataset used as query and subject database) on mouse representative ORF databases separately to detect homology. An E-value of 1×10^-10^ was used in the BLAST search at the Ensembl (http://www.ensembl.org/index.html). Individual BLAST hits found for the same pair of ORFs were combined in the following way: when two ORFs have multiple BLAST hits with overlapping alignable regions, a non-overlapping combination of those hits with the longest alignable regions was selected out of all possible combinations. This combination was then treated as a single BLAST hit. Next the BLAST hits were filtered to keep only those with at least 30% identity, bitscore of at least 50, aligning at least 50% of the length of both ORFs, and excluding self-hits. Then duplicates, or multi-loci genes, were defined as pairs of ORFs having two-way hits in the filtered set of BLAST hits. Thus all ORFs were classified as singletons or duplicates. Gene families were then identified using single-linkage clustering: Step 1. Initially all genes are in their own families. Step 2. When two genes A and B are found to have a two-way hit, their whole families are merged together. Step 3. Repeat step 2 until no further merging can be done.

Additionally, to investigate the presence in mammalian genomes of alternative splicing SMO isoforms, a search in the public databases was performed based on exon VIa sequence annotation [10].

### Phylogenic analysis

Phylogenetic analyses were performed by the Maximum Likelihood and the Bayesian inference methods using the amino acid sequences with the yeast as outgroup.

Maximum Likelihood analysis was carried out in PALM [36], which incorporates several programs in an integrated framework for phylogenetic reconstruction with automatic likelihood model selectors. Multiple amino acidic sequences alignment was performed by Clustal W [37]. The fitness among 112 models of protein evolution was estimated trough PhyML/ProtTest [38,39] and the optimal model was selected under the Bayesian Information criterion (BIC). The JTT model with a proportion of invariable sites (I) and gamma-distributed rates across sites (G) was selected for the phylogenetic inference performed via PhyML. Nodes support for the resulting phylogenetic tree was evaluated by 500 bootstrap replications.

Bayesian inference analysis was performed with MrBayes 3.2 [40] under the JTT model of protein evolution with gamma-distributed rates across sites and a proportion of invariable sites (rates = invgamma) suggested by ProtTest. Two independent runs of four Markov chain Monte Carlo (MCMC) chains each were executed in parallel for two million generations, sampling every 100 generations. Posterior probabilities for nodes were derived from a majority rule consensus of the trees sampled after convergence (25% was setted as burnin for sumt and sump).

### Homology modeling and identification of critical amino acids

The multiple alignment of the retrieved PAO sequences, obtained using Clustal W 2.0 [41], was visualized using Jalview alignment editor [32] (Additional file 1: Figure S1).

The Jalview tool, which allows mapping of the sequence conservation data onto the three-dimensional structure of a reference protein, was used to generate a three-dimensional view of the sequence conservation data in the active site region of SMOs and APAOs, using previously published molecular models of the mouse enzymes [11,12].

The structural model of *Drosophila melanogaster* PAO (DmPAO) has been built by homology using the three dimensional structure of maize PAO (ZmPAO; PDB code 1B5Q) [31], the closest homologue found in the Protein Data Bank, as a template. In detail, the template structure was chosen through two PSI-Blast [42] iterations against the PDB sequence database using the sequence coded Swiss-Prot Q9VHN8 (corresponding to DmPAO) as a bait. Pairwise sequence alignment between DmPAO and ZmPAO was extracted from a multiple sequence alignment of all known PAO sequences obtained using Clustal W 2.0. Based on this alignment, the structural model of DmPAO was built using the homology modeling program Nest [43]. The ‘alignment tuning’ option of nest was used to refine the sequence alignment, to avoid the unlikely occurrence of insertions and deletions within secondary structure elements.

## Abbreviations

APAO: Acetylpolyamine oxidase; AS: Alternative splicing; FMS1: *Saccharomyces cerevisiae* polyamine oxidase protein; NDA: Nuclear Domain A; NDB: Nuclear Domain B; PA: Polyamines; PAO: Polyamine oxidase; SMO: Spermine oxidase.

## Authors’ contribution

This work was planned by FP and PM and carried out in collaboration with DS and MC. DS performed the phylogenetic analyses and participated in discussing the evolutionary aspects and drafting the manuscript. RA critically revised the manuscript for important intellectual content and data analysis. All authors read and approved the final manuscript.

## Supplementary Material

Additional file 1 **Figure S1. **Amino acid sequence alignment of selected polyamine oxidases. The amino acid blocks are coloured according to BLOSUM62 score (dark blocks corresponding to higher score). Residues building up the active site are indicated by red stars, those forming the putative SMOs specificity pocket (Glu216 and Ser218 in mouse SMO) are indicated by green stars. For acronyms and isoform numbering see Tables 1 and 2. The amino acid sequence of the *Zea mays* PAO (ZmPAO) whose structure has been used for comparative modeling of the DmPAO is also shown for reference. The figure was generated using Jalview [32]. Click here for file
