# Supplementary figures and images for: Molecular evolution of the polyamine oxidase gene family in Metazoa
Source: BMC Evol Biol. 2012 Jun 20;12:90. doi: 10.1186/1471-2148-12-90 (PMC3517346; doi:10.1186/1471-2148-12-90)

**Figure S1**


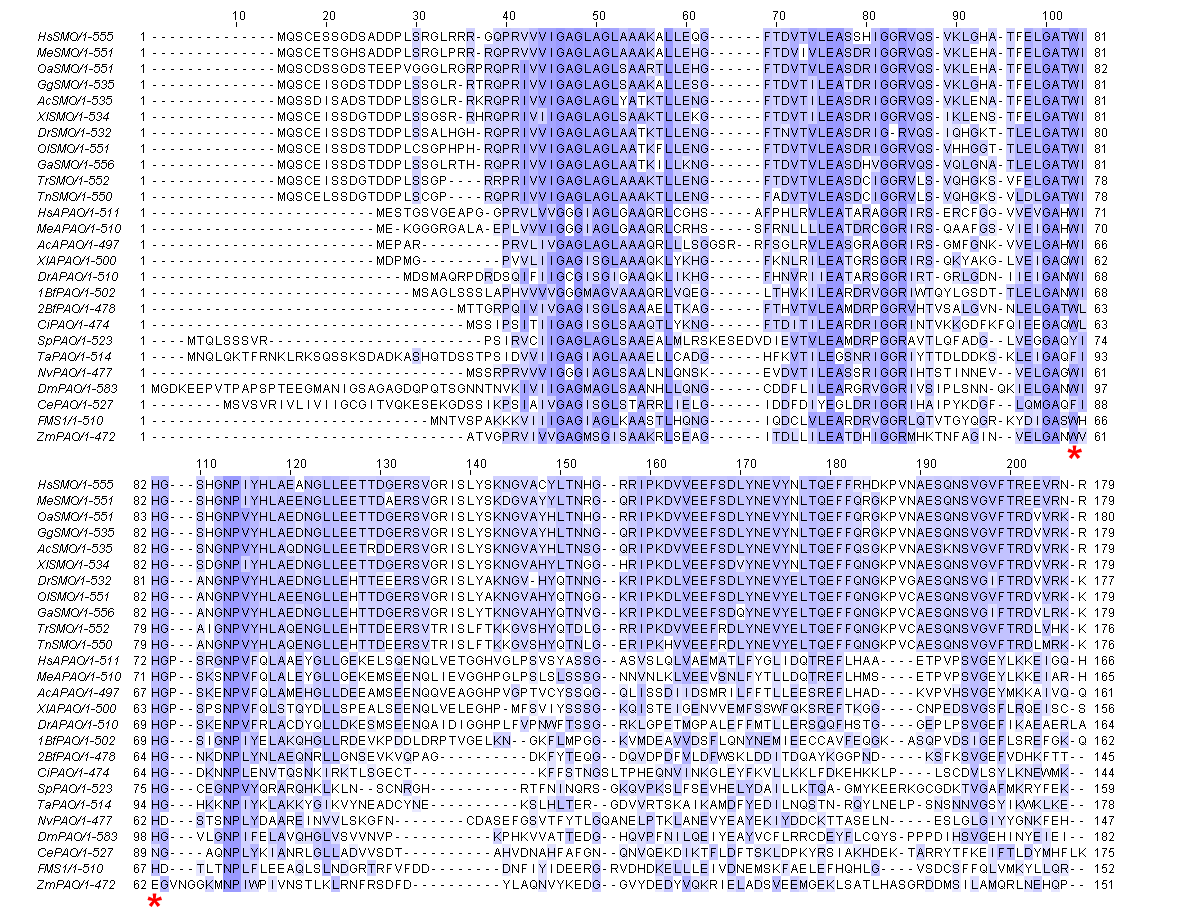


**Figure S1 (continues)**


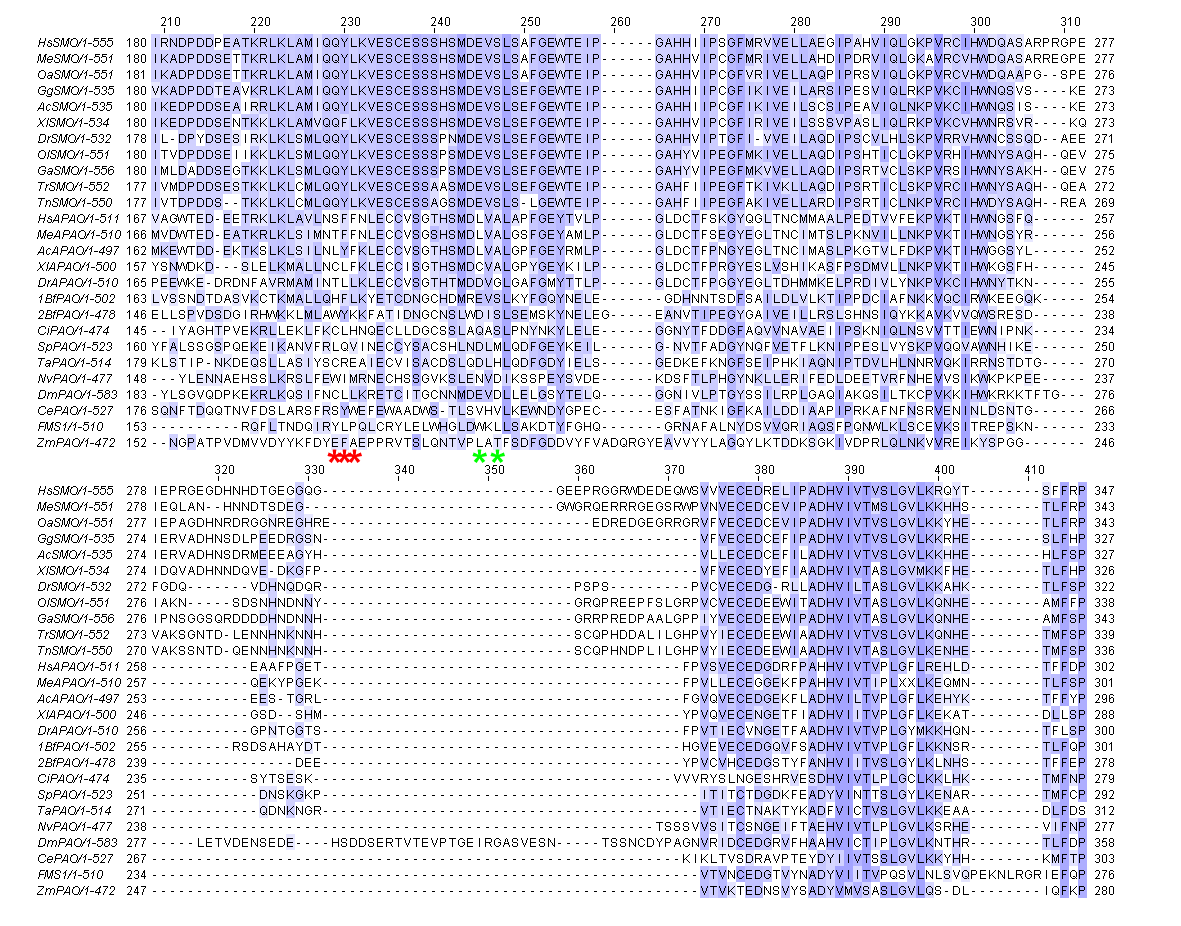


**Figure S1 (continues)**


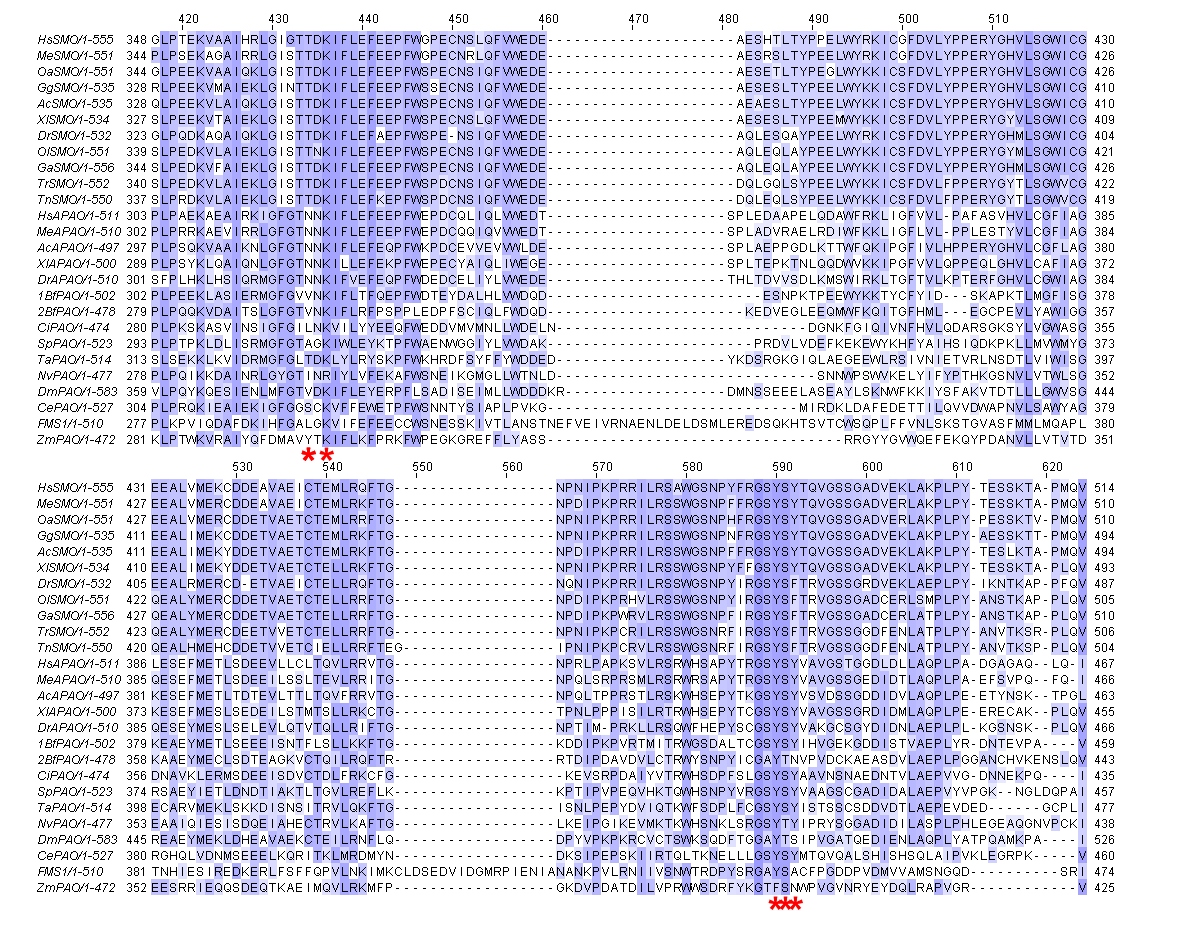


**Figure S1 (continues)**


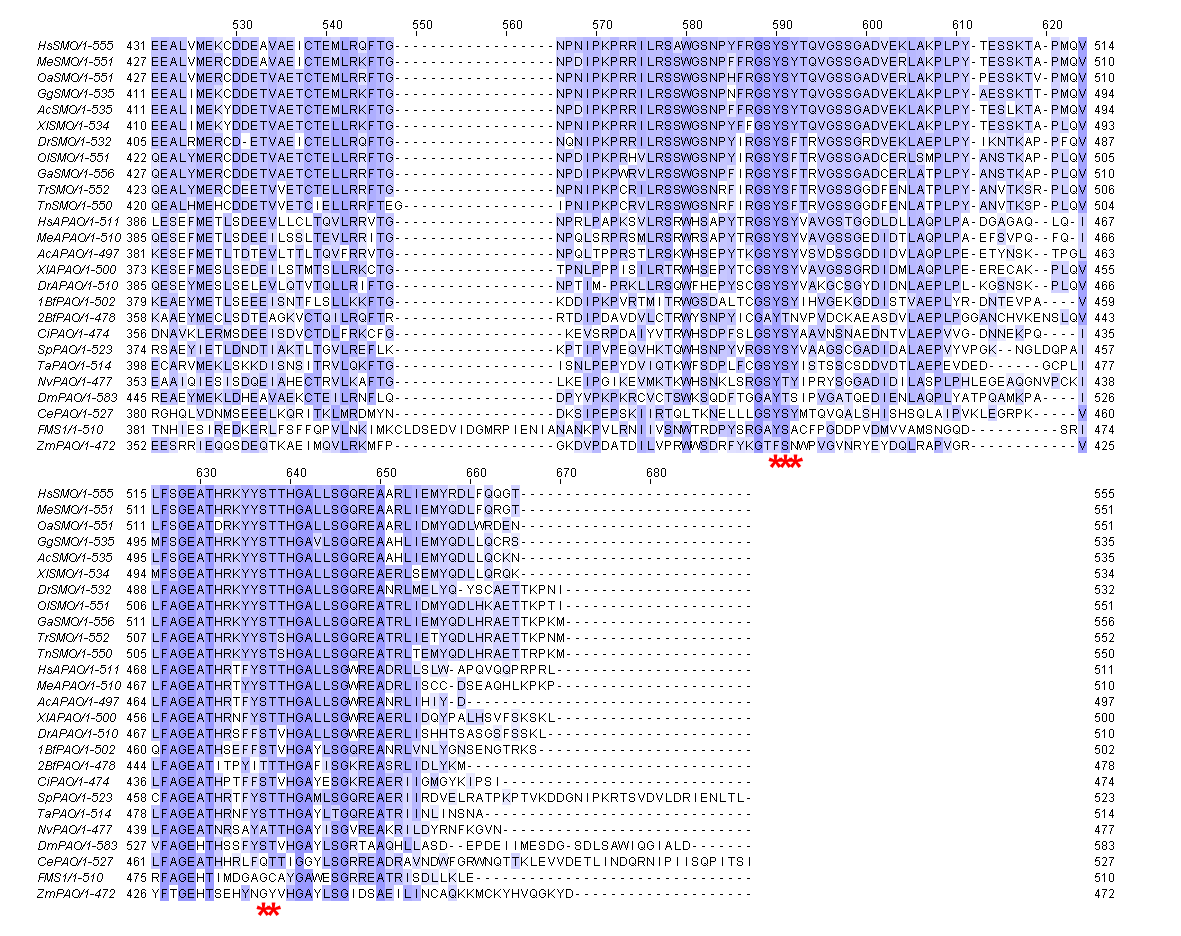

Supplement: Additional file 1 — Figure S1. Amino acid sequence alignment of selected polyamine oxidases. The amino acid blocks are coloured according to BLOSUM62 score (dark blocks corresponding to higher score). Residues building up the active site are indicated by red stars, those forming the putative SMOs specificity pocket (Glu216 and Ser218 in mouse SMO) are indicated by green stars. For acronyms and isoform numbering see Tables 1 and 2. The amino acid sequence of the Zea mays PAO (ZmPAO) whose structure has been used for comparative modeling of the DmPAO is also shown for reference. The figure was generated using Jalview [32]. [file 1471-2148-12-90-S1.doc]
